# Hematopoietic Stem Cells in Neonates: Any Differences between Very Preterm and Term Neonates?

**DOI:** 10.1371/journal.pone.0106717

**Published:** 2014-09-02

**Authors:** Lukas Wisgrill, Simone Schüller, Markus Bammer, Angelika Berger, Arnold Pollak, Teja Falk Radke, Gesine Kögler, Andreas Spittler, Hanns Helmer, Peter Husslein, Ludwig Gortner

**Affiliations:** 1 Dept. of Pediatrics and Adolescent Medicine, Division of Neonatology, Paediatric Intensive Care & Neuropaediatrics, Medical University of Vienna, Vienna, Austria; 2 Institute for Transplantation Diagnostics and Cell Therapeutics, Heinrich Heine University Medical Center, Duesseldorf, Germany; 3 Department of Surgery, Research Labs & Core Facility Flow Cytometry, Medical University of Vienna, Vienna, Austria; 4 Dept. of Obstetrics and Gynecology, Medical University of Vienna, Vienna, Austria; 5 Dept. of Pediatrics and Neonatology, Saarland University, Homburg, Saar, Germany; Vanderbilt University, United States of America

## Abstract

**Background:**

In the last decades, human full-term cord blood was extensively investigated as a potential source of hematopoietic stem and progenitor cells (HSPCs). Despite the growing interest of regenerative therapies in preterm neonates, only little is known about the biological function of HSPCs from early preterm neonates under different perinatal conditions. Therefore, we investigated the concentration, the clonogenic capacity and the influence of obstetric/perinatal complications and maternal history on HSPC subsets in preterm and term cord blood.

**Methods:**

CD34+ HSPC subsets in UCB of 30 preterm and 30 term infants were evaluated by flow cytometry. Clonogenic assays suitable for detection of the proliferative potential of HSPCs were conducted. Furthermore, we analyzed the clonogenic potential of isolated HSPCs according to the stem cell marker CD133 and aldehyde dehydrogenase (ALDH) activity.

**Results:**

Preterm cord blood contained a significantly higher concentration of circulating CD34+ HSPCs, especially primitive progenitors, than term cord blood. The clonogenic capacity of HSPCs was enhanced in preterm cord blood. Using univariate analysis, the number and clonogenic potential of circulating UCB HSPCs was influenced by gestational age, birth weight and maternal age. Multivariate analysis showed that main factors that significantly influenced the HSPC count were maternal age, gestational age and white blood cell count. Further, only gestational age significantly influenced the clonogenic potential of UCB HSPCs. Finally, isolated CD34+/CD133+, CD34+/CD133– and ALDH^high^ HSPC obtained from preterm cord blood showed a significantly higher clonogenic potential compared to term cord blood.

**Conclusion:**

We demonstrate that preterm cord blood exhibits a higher HSPC concentration and increased clonogenic capacity compared to term neonates. These data may imply an emerging use of HSPCs in autologous stem cell therapy in preterm neonates.

## Introduction

Umbilical cord blood (UCB) is a rich source of hematopoietic stem and progenitor cells (HSPCs). During the past decades, human cord blood of term neonates has been established as a potential source for HSPC transplantation [1]. Therefore, many studies focus on hematopoietic and immunological features of HSPCs in term newborns.

In normal human development, fetuses gradually mature to adapt to extrauterine conditions. Premature birth is often associated with obstetric and perinatal complications, interrupting the physiologic development process and resulting in organ damage or dysfunction. The role of HSPCs in physiologic and pathophysiologic human development still remains uncertain. Hematopoietic stem and progenitor cells, as assessed by the expression of CD34, are capable of differentiating into non- hematopoietic cells such as microglia [2], hepatocytes [3], and type II alveolar pneumocytes [4]. These findings may indicate a supporting role of HSPCs in the intrauterine development.

The utilization of term UCB has widely become an easily available and acceptable alternative for stem cell transplantation of hematological and non-hematological disorders. Preterm birth is a major determinant of neonatal mortality and morbidity and is associated with severe complications including bronchopulmonary dysplasia (BPD), white matter injury and intracranial hemorrhage [5]. In the last decades, the potential of non-oncologic stem cell and mononuclear cell therapies have been investigated for the regeneration of impaired organ development and tissue regeneration [6]–[10]. In clinical settings, the infusion of autologous UCB in infants with neurologic disorders seems feasible and safe [11], [12]. Double-blind randomized studies are needed to evaluate the therapeutic benefit of autologous UCB transfusion in neonates.

Despite the growing interest of regenerative medicine in preterm neonates [13], less is known about the biological properties of HSPCs obtained from preterm cord blood (PCB). Thus, we aimed to investigate the number and clonogenic capacity of circulating CD34+ HSPCs subsets in PCB and term cord blood (TCB) and the influence of obstetric and maternal history on these subsets. Further, we determine the clonogenic capacity of isolated HSPC subsets of PCB and TCB.

## Materials and Methods

### Study population

Sixty newborns were enrolled in the study between February and August 2013. Very preterm infants (n = 30; 24–32 weeks of gestational age (GA)) were compared with term newborns (n = 30; 38–42 weeks of GA). The study was approved by the ethics committee of the Medical University of Vienna and written informed consent was obtained from the parents before birth. Heparinized whole blood obtained from umbilical cord was collected immediately after cesarean section and processed within six hours after collection. Maternal history as well perinatal and neonatal variables were documented until discharge from hospital.

### Definition of clinical parameters

Prolonged rupture of membranes (PROM) was defined as rupture of membranes ≥18 h prior to delivery. Chorioamnionitis was defined as described previously [14], based on the presence of maternal fever ≥38°C with two or more of the following criteria: maternal leucocytosis (>15 000 cells/mm^3^), maternal (>100 beats/min) or fetal (>160 beats/min) tachycardia, uterine tenderness and/or foul odor of the amniotic fluid. Early labour was defined as onset of labour before caesarean section. Small-for-gestational-age (SGA) was defined as birth weight below the 10th percentile using Fenton growth charts [15]. Antenatal corticosteroid administration was defined as any intramuscular administration of betamethasone ≥24 h prior to delivery. Tocolysis was defined by the administration of the oxytocin antagonist Atosiban or the β_2_ adrenergic agonist Hexoprenalin within 12 hours of delivery. Gestational diabetes mellitus (GDM) was diagnosed by blood sugar levels of >126 mg/dl after fasting, or a pathological oral glucose tolerance test. Preeclampsia was defined by high maternal blood pressure (>140 mmHg systolic or >90 mmHg diastolic) and proteinuria (>300 mg/24 h).

### Enumeration of umbilical cord HSPCs

The enumeration of HSPC was conducted in whole UCB using BD Trucount tubes (BD Biosciences, San Jose, CA, USA), according to the manufacturer’s instruction. 100 µl whole UCB were added by reverse pipetting to BD Trucount tubes and stained with anti- CD45− FITC, anti- CD34− PE, anti- CD38− PE- Cy7, anti- CD10− PE- CF594, 7-AAD (all BD Biosciences) and anti- CD133– APC (Miltenyi Biotechnology, Bergisch, Gladbach, Germany). After immunofluorescence staining, erythrocytes were lysed with ammonium chloride lysis solution and analyzed within one hour by flow cytometry (LSRII, BD Biosciences) and analysed using FlowJo software (TreeStar Inc., Ashland, OR, USA). For enumeration of HSPC in UCB, CD34+ cells were gated according to the modified ISHAGE criteria (CD45^dim^/7-AAD-/CD34+ cells). CD10 was added to exclude B- lymphoid progenitors. The number of cells/µl was calculated as [(# gated cells/# acquired beads) * (# of beads per test/test volume).

### Progenitor cell isolation according to common stem cell markers or aldehyde dehydrogenase activity

UCB mononuclear cells (MNCs) were isolated from fresh heparinized cord blood by Ficoll gradient centrifugation (Ficoll-Paque PLUS; Amersham, GE Healthcare Life Sciences, Little Chalfont, Buckinghamshire, UK) and remaining erythrocytes were lysed with ammonium chloride lysis solution (BD Biosciences). 1×10^6^ MNCs were subsequently stained with anti- CD45− FITC, anti- CD34− PE, 7-AAD (BD Biosciences) and anti- CD133– APC (Miltenyi Biotec) for isolation of CD34+/CD133+ and CD34+/CD133– HSC subpopulations.

Another MNC aliquot was assayed for aldehyde dehydrogenase (ALDH) activity using Aldefluor reagent (StemCell Technologies, Marseille, France) according to the manufacturer’s instructions. Isolated cells were resuspended in aldefluor assay buffer and an appropriate amount of aldefluor substrate was added to 1×10^6^ MNCs. Cells were incubated for 30 minutes at 37°C in a waterbath for conversion of substrate to a fluorescent product. An aliquot of Aldefluor- stained cells were immediately treated with diethylaminobenzaldehyde (DEAB), a specific ALDH inhibitor, to serve as negative control. For evaluating clonogenic capacity, SSC^low^/ALDH^high^ and SSC^low^/ALDH^low^ cells were selected by FACS. For all samples, 400 cells were directly sorted into 100 µl IMDM using a MoFlo Astrios flow cytometer cell sorter (Beckman Coulter, Brea, CA, USA). For phenotypic analysis of SSC^low^/ALDH^high^ cells, aldefluor- substrate labeled MNCs were costained with anti- CD34 PE and anti- CD133 APC (Miltenyi Biotechnology) and subsequently analyzed on a LSR II (BD Biosciences).

### Clonogenic progenitor assay

Purified HSC population were resuspended into 900 µl semisolid methylcellulose medium (Methocult H4334, StemCell Technologies) and plated in triplicates on 24- well plates with a seeding density of 100 HSPC/well. For whole blood progenitor assays, 100 µl UCB was lysed with ammonium chloride lysis solution, centrifuged and resuspended in IMDM. An appropriate amount of lysed whole blood was added to 900 µl methylcellulose medium and plated in triplicates reaching a seeding density of 100 HSPC/well. Cells were incubated in humidified atmosphere (37°C/5% CO_2_) for 14 days and colonies were evaluated for number and morphology by light microscopy.

### Statistical analysis

Statistical analysis was performed with IBM SPSS Statistics 21. Kolmogorov- Smirnov test was performed to prove normal distribution. Student’s T- Test was used to analyze normal distributed data. In case of non- normal distribution, data were further analyzed using Mann- Whitney- U test. Bonferroni correction for multiple testing was used. Differences of quantitative variables were compared by Mann- Whitney U test and qualitative variables with Fisher’s exact test. Correlation between variables was determined using Spearman’s rank correlation coefficient. Multiple linear backward regression was performed to evaluate the effects of obstetric, perinatal and neonatal factors on the UCB HSC count. A p<0.05 was considered as statistically significant.

## Results

### Clinical characteristics of preterm and term newborns

Obstetric history as well perinatal and neonatal clinical variables of preterm and term newborns are summarized in Table 1. Mean difference in GA between preterm and term neonates was 9.53 weeks. Preterm neonates had a significantly lower mean birth weight (1354.77±553.80 g vs. 3392.77±489.06 g) including 10 infants of extremely low birth weight (ELBW) (755.80±163.61 g), 7 of very low birth weight (VLBW) (1224.14±113.45 g), and 13 of low birth weight (LBW) (1885.85±316.56 g) compared to term newborns (3392.77±489.06 g). Early labour, chorioamnionitis, tocolysis, antenatal steroids, PROM and APGAR scores at 5 and 10 min were statistically significantly different between both groups, whereas maternal age, number of pregnancies, GDM, preeclampsia, SGA and smoking were not statistically different. Both groups were matched by gender. The most abundant obstetric complication in the preterm group was PROM (46.7%) followed by chorioamnionitis (16.7%) and preeclampsia (13.3%). One preterm neonate was diagnosed with meconium peritonitis.

**Table 1:** Obstretic, perinatal and neonatal clinical parameters.

Parameter	Preterm newborns (n = 30)	Term newborns (n = 30)	p-value
Maternal age	32.73±7.09 (32)	34.03±6.48 (33.5)	0.403
No. of Gravidity	2.20±2.79 (1)	2.17±0.98 (2)	0.189
No. of Parity	1.57±0.81 (1)	1.8±0.84 (2)	0.619
Maternal diabetes	3 (10%)	5 (16.7%)	0.706
Smoking	1 (3.3%)	4 (13.3%)	0.353
Preeclampsia	4 (13.3%)	0 (0%)	0.112
Chorioamnionitis	9 (30%)	0 (0%)	0.002
Gestational age (wks)	29.63±2.70 (30.14)	39.16±1.17 (38.92)	<0.0001
Birth weight (g)	1354.77±553,80 (1318)	3392.77±489.06 (3360)	<0.0001
SGA	5 (16.7%)	0 (0%)	0.052
Male Gender	16 (53.3%)	19 (63.3%)	0.601
5- min APGAR	8.47±0.93 (8)	9.87±0.43 (10)	<0.0001
10- min APGAR	8.87±0.90 (9)	9.97±0.18 (10)	<0.0001
Antenatal corticosteroids	27 (90%)	0 (0%)	<0.0001
Labour before cesareansection	13 (43.3%)	2 (6.7%)	0.002
Tocolysis	13 (43.3%)	0 (0%)	<0.0001
PROM	14 (46.7%)	0 (0%)	<0.0001

Data are shown as mean ± SD (median) or n (%), SGA: small-for-gestational-age; PROM: prolonged rupture of membranes.

### Preterm newborns display a higher number of circulating cord blood CD34+ HSPC than term newborns

**Figure 1:**
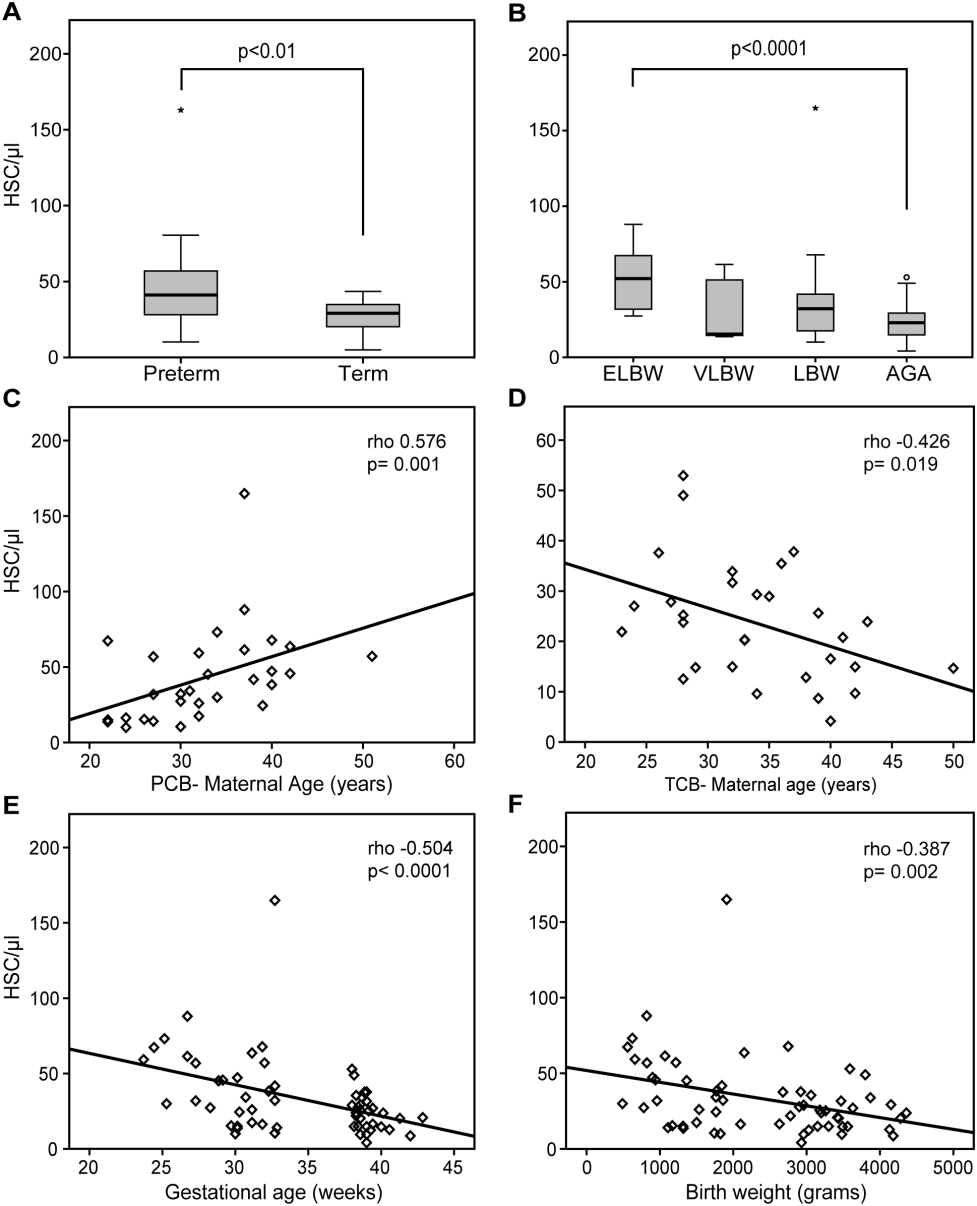
HSPC count and correlation with clinical parameters. Number of circulating HSPCs in umbilical cord blood of preterm and term neonates (A) and in ELBW, VLBW, LBW and appropriate for gestational age (AGA) infants (B). Correlation between number of HSPCs and maternal age of PCB (C) and TCB (D), gestational age (E) and birth weight (F).

The number of WBC/ml in UCB was significantly higher in TCB than in PCB. However, the concentration of circulating CD34+ HSPCs in UCB was significantly higher in PCB than in TCB (Figure 1A). The proportion of CD34+/CD133– HSPCs was significantly higher in PCB than in TCB, whereas the proportion of CD34+/CD133+ HSPCs was higher in TCB. Furthermore, PCB showed a higher percentage of CD34+/CD38– HSPCs compared to TCB (Table 2). The percentage of CD34+/CD38−/CD133+ HSPCs was not significantly higher in PCB than TCB. CD34+ HSPC count was not statistically different between ELBW, VLBW and LBW infants, but was higher in ELBW infants compared to term newborns (Figure 1B). In the univariate analysis maternal age, gestational age and birth weight significantly correlated with the number of CD34+ HSPCs in both groups. Maternal age positively correlated with circulating CD34+ HSPCs in PCB (Fig. 1C), whereas significant inverse correlations were found in TCB (Fig. 1D). Furthermore, GA (Fig. 1E) and birth weight (Fig. 1F) negatively correlated with CD34+ HSPC in UCB. The percentage of CD34+/CD133+ cells in CB positively correlated with GA and birth weight of infants. Multiple linear backward regression showed that main factors that significantly influence the HSPC count were maternal age (p<0.01), gestational age (p<0.001) and white blood cell count (p<0.0001).

**Table 2:** HSPC subsets and WBC count of preterm and term cord blood.

	PCB (n = 30)	TCB (n = 30)	p-value
Number of CD45+ WBC (×10^6^/ml)	6.5±3.1	9.4±2.8	<0.0001
Number of CD34+ (×10^4^/ml)	4.3±3.1	2.3±1.1	<0.01
Percent of CD34+/CD38+/CD133+ (%)	62.18±10.21	73.14±5.68	<0.0001
Percent of CD34+/CD38+/CD133– (%)	37.82±10.21	26.86±5.68	<0.0001
Percent of CD34+/CD38– (%)	17.08±6.52	12.78±6.18	0.008
Percent of CD34+/CD38−/CD133+ (%)	80.32±12.10	70.38±22.76	0.051
Percent of CD34+/CD38−/CD133– (%)	19.68±12.10	29.62±22.76	0.051

PCB: preterm cord blood; TCB: term cord blood; WBC: white blood cells; HSPC: hematopoietic stem and progenitor cells.

### Higher in- Vitro clonogenic capacity of PCB derived HSPCs compared to TCB

**Figure 2:**
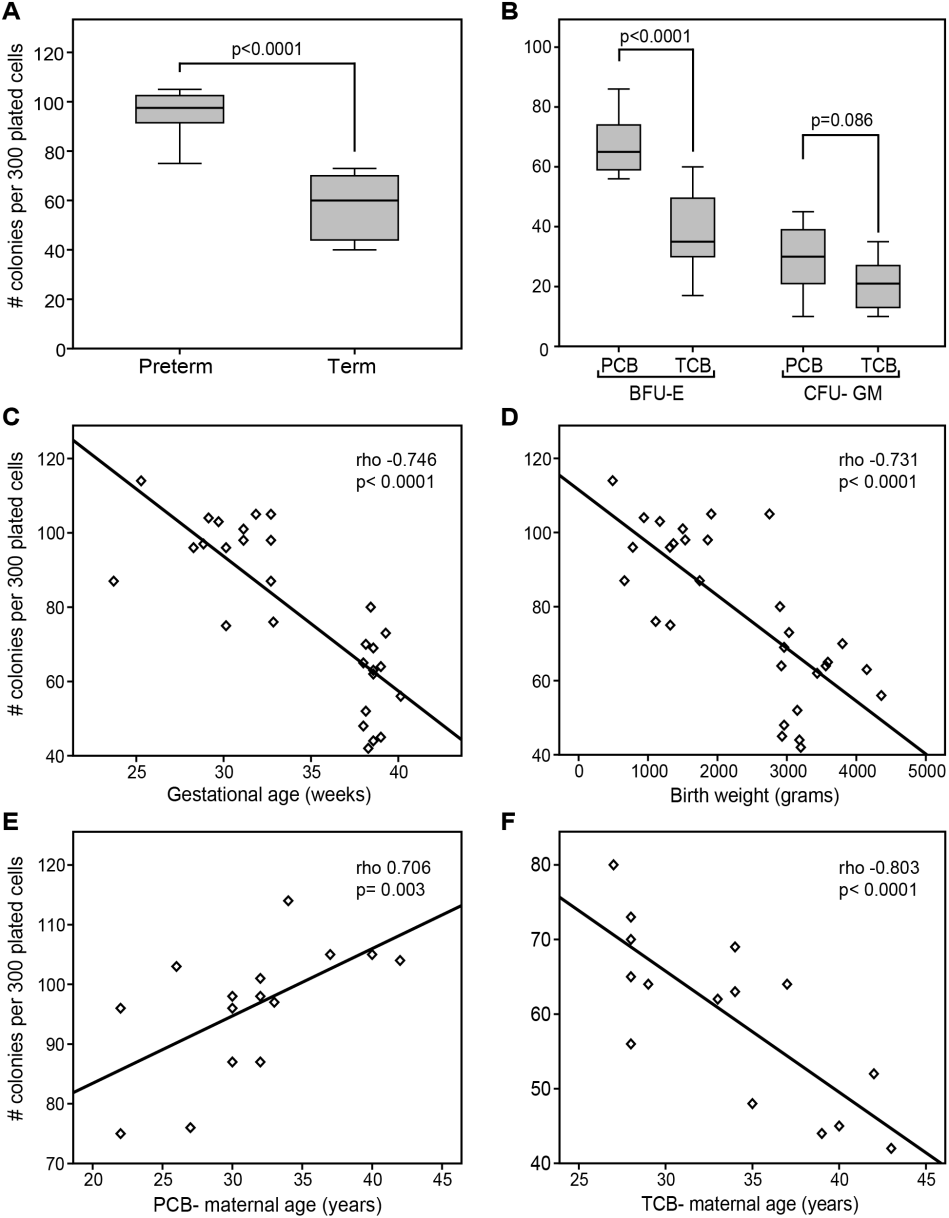
Clonogenic capacity and correlation with clinical parameter. Total clonogenic capacity of PCB and TCB (A) and potential to differentiate into CFU- GM and BFU-E (B). Correlation between total number of colonies and gestational age (C), birth weight (D), maternal age of PCB (E) and TCB (F). Data are presented as number of colonies per 300 plated HSPCs.

In our lysed whole blood clonogenic progenitor assay (LWBA), the clonogenic capacity of CD34+ HSPCs was higher in PCB than TCB (Fig. 2A). The number of erythrocyte burst- forming units (BFU- E) was significantly higher in PCB than in TCB. However, there was no statistically significant difference in the number of granulocyte-macrophage colony-forming units (CFU-GM) of PCB and TCB (Fig. 2B) (Table 3). In the univariate analysis gestational age, birth weight and maternal age correlated with the clonogenic capacity of UCB HSPCs. GA (Fig. 2C) and birth weight (Fig. 2D) negatively correlated with the total number of colonies and BFU- E. Furthermore, maternal age positively correlated with clonogenic capacity in PCB (Fig. 2E), whereas significant inverse correlations were found in TCB (Fig. 2F). Interestingly, the number of BFU-E in PCB (rho 0.755; p = 0.001) also positively correlated with the maternal age. Using multiple linear backward regressions, only gestational age (p<0.012) significantly influenced the clonogenic capacity of UCB HSPCs.

**Table 3:** Clonogenic capacity of HSPCs of preterm and term cord blood.

		PCB	TCB	p-value
**LWBA**	No. of colonies	96.13±10.81	59.80±11.49	<0.0001
	No. of BFU- E	67.27±10.05	38.33±11.97	<0.0001
	No. of CFU- GM	28.87±12.11	21.47±8.21	0.086
**CD34+/CD133+**	No. of colonies	101.33±3.51	51.67±16.50	0.007
	No. of BFU- E	58.00±7.54	31.67±7.64	0.013
	No. of CFU- GM	43.34±6.43	20.00±8.89	0.021
**CD34+/CD133–**	No. of colonies	57.00±6.00	21.33±4.51	0.001
	No. of BFU- E	44.67±4.16	14.33±2.08	<0.0001
	No. of CFU- GM	12.33±4.16	7.00±2.65	0.134
**ALDH^high^**	No. of colonies	85.34±5.51	53.34±7.09	0.004
	No. of BFU- E	61.00±5.00	35.00±5.57	0.004
	No. of CFU- GM	24.34±5.03	18.34±1.53	0.119

Clonogenic potential of HSCs in the lysed whole blood assay (LWBA, n = 15) and sorted HSC subpopulations (n = 3).

### Isolated CD34+/CD133+, CD34+/CD133– and ALDH^high^ HSPCs of PCB exhibit higher clonogenic capacity than in TCB

**Figure 3:**
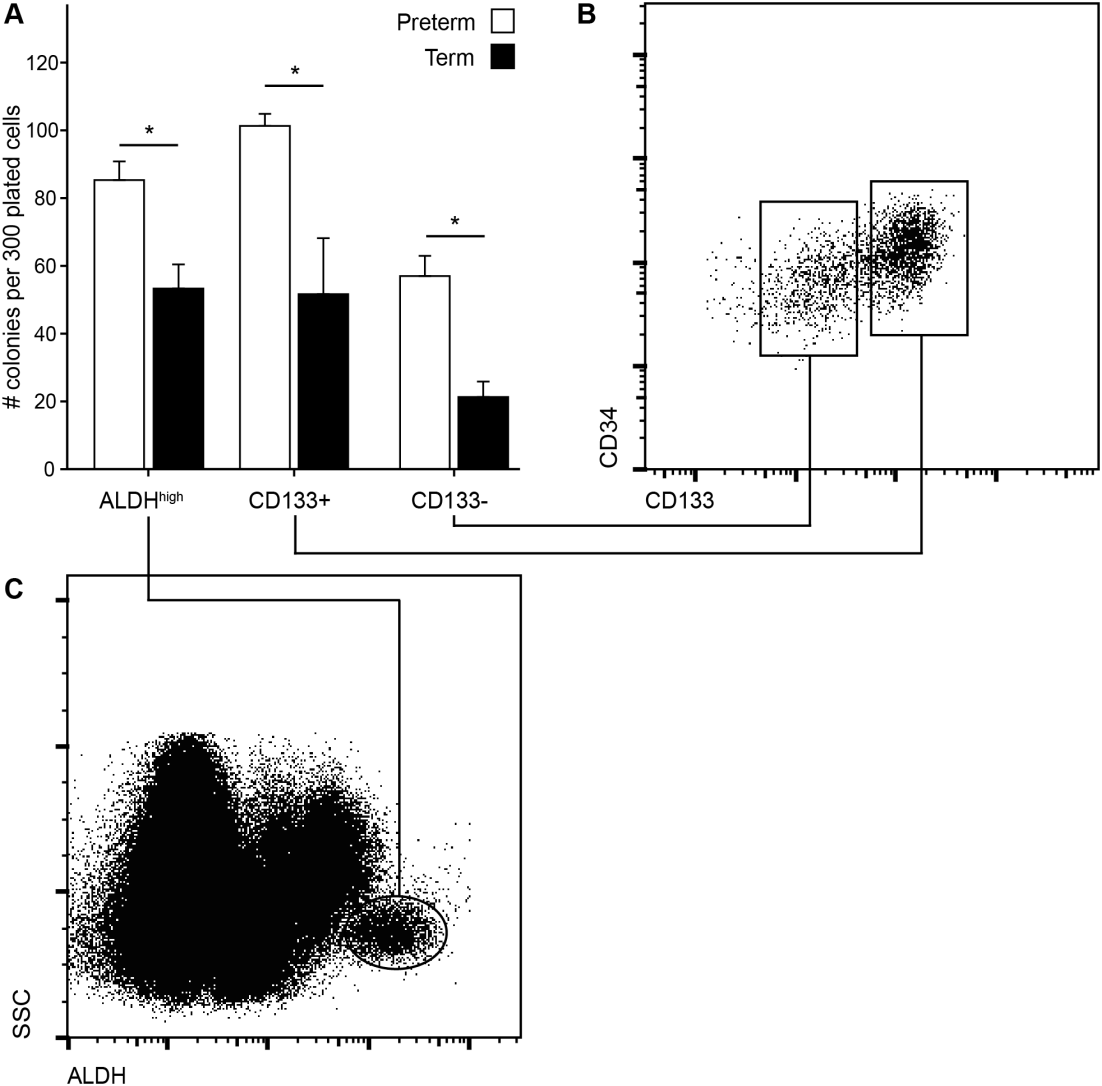
Clonogenic capacity of isolated HSPC subsets. Isolated HSPCs from preterm neonates displayed higher clonogenic capacity compared to term neonates (A). UCB MNCs were either stained to stem cell markers CD34 and CD133 (B) or ALDH activity (C). Data are presented as number of colonies per 300 plated HSPCs.

To evaluate the higher clonogenic capacity of PCB, we isolated HSPCs according to common stem cell markers (CD34+/CD133+, CD34+/CD133–) and ALDH activity (Fig. 3). Isolated CD34+/CD133+ HSPCs of PCB displayed a significant higher clonogenic capacity compared to TCB. The percentage of CFU- GM and BFU- E did not differ significantly between PCBs and TCBs. Furthermore, CD34+/CD133– HSPCs exhibited a higher clonogenic capacity when isolated from PCB as opposed to TCB. Interestingly, CD34+/CD133– HSPCs had a higher potential to differentiate into BFU- E than CFU- GM in both groups. UCB HSPCs sorted according to their ALDH activity displayed a similar clonogenic capacity and BFU-E/CFU- GM differentiation pattern as the lysed whole blood assay. The clonogenic capacity of ALDH^high^ HSPCs was higher in PCB than TCB. ALDH^high^ cells of PCB and TCB showed a similar proportion of CD133+ HSPCs. ALDH^low^ cells, serving as negative control, showed hardly any clonogenic potential in both groups. Although the number of BFU- E was significantly higher in PCB than in TCB, there was no significant difference in the number of CFU- GM (Table 3).

## Discussion

Many other studies examined the impact of obstetric, perinatal and neonatal factors in term newborns, showing an influence of gestational age, birth weight, maternal age, small for-gestational-age, preeclampsia, delivery mode and fetal distress [16]–[23]. However, those studies investigated interfering factors on term cord blood HSPC count. In our study, we found differences in CD34+ HSPC concentrations and in vitro clonogenic capacity in preterm compared with term cord blood cells and, using multiple linear backward regression, three predictive factors influencing the count and in vitro clonogenic capacity: (1) gestational age, (2) white blood cell count and (3) maternal age.

The gestational age of neonates is a consistent predictor for both HSPC concentration and clonogenic capacity. Gasparoni et al. [18] reported a gestational age-dependent decrease of concentration and clonogenic potential of CD34+ HSPCs, which is in line with our results. The HSPC concentration in preterm cord blood samples might be influenced by individual occurring fetal stress during premature birth. Acute fetal stress seems to induce the release of cytokines resulting in higher HSPC counts [22], [23]. An approach to explain the higher clonogenic potential of HSPCs from PCB might be the fluctuating cytokine and chemokine levels in neonates that reflect the transfer of hematopoiesis from liver to bone marrow, which may vary in certain preterm newborns [17].

Cord blood from term newborns exhibit a higher WBC count than those from preterm newborns [24]. Although TCB possesses a higher WBC count, we measured a fewer HSPC concentration compared to preterm cord blood, which is in contrast to other studies [21], [25]. This might be explained by different used enumeration methods. In our study, we used a lyse-no-wash whole blood assay without prior cell separation or enrichment step minimizing cell loss.

To date, the influence of maternal age on cord blood HSPCs is disputable. As described by others [16], [24], [26], maternal age had no impact on the HSPC concentration. In contrast, other groups reported an influence of maternal age on HSPC concentration, which is in line with our results [19], [27]. In addition, the univariate analysis shows a maternal age-dependent influence on the clonogenic capacity. The etiology for this finding is unclear. Speculative hypotheses include alternating fetal hormone levels during pregnancy [28]. Interestingly, we found no clinical correlation of premature birth associated morbidities on HSPC count and clonogenic capacity. In our study, preeclampsia and small-for-gestational-age did not affect the cord blood HSPC concentration as described by other groups [20], [29] and may be due to our small sample size. Although tocolysis with Atosiban had no effect on the HSPC population, the influence of magnesium sulfate on CD34+ cells, which is widely used as tocolytic drug in the U.S., is still unknown.

In 1997, Yin et al. [30] identified the novel stem cell marker CD133 restricted to a subset of CD34+ HSPC with long-term repopulating ability [31]. The indicated role of HSPC subsets in tissue repair and the sufficient isolation from UCB may suggest a therapeutic capacity of different HSPC subsets in regenerative medicine. Taguchi et al. [32] showed that the systemic administration of CD34+ HSPCs promote the neovascularization and enhance the neurogenesis in a mouse stroke model. Especially, CD133+ HSPCs exhibit a high potential to regenerate ischemic tissue by promoting local angiogenesis [33], [34]. They are considered to possess both hematopoietic and endothelial lineage differentiation potential [35]. The potential use of CD133+ HSPCs in regenerative stem cell therapy is under investigation [36], [37]. Although CD34+/CD133+ HSPCs from preterm cord blood showed higher clonogenic potential, the regenerative potential needs to be evaluated. The capability of CD133– HSPCs, especially from preterm cord blood, for regenerative purposes is still unknown. In both groups, CD34+/CD133– HSPCs had a lower clonogenic capacity, which is in consent with other studies [30], [38].

Isolating UCB HSPCs according to high ALDH activity has been an efficient and reproducible method without excessive cell manipulation. As reported by Hess et al., ALDH^high^ HSPCs from TCB demonstrated reliable NOD/SCID repopulating function [39]. Furthermore, UCB ALDH^high^ cells are considered to be proangiogenic progenitors that integrate into ischemic tissue and promote vascular regeneration [40]. Isolated HSPCs according to their enhanced ALDH activity in PCB demonstrated a HSPC phenotype and higher in-vitro clonogenic potential than in TCB. Our data indicate that HSPCs can be effectively isolated from PCB using ALDH activity.

Umbilical cord blood is a robust source of different stem and progenitor cells with lower risk of severe acute graft versus host disease when used for allogeneic transplant [41]. Despite the higher CD34+ HSPC counts in PCB, some UCB samples of preterm neonates displayed a lower HSPC concentration compared to term neonates. Another critical point is the lower obtainable blood volume from preterm cord blood. The mean volume of the collected PCB samples (n = 6; mean GA 29±1.5 weeks; range 27+1–31+1 weeks) was 15.5±7.8 ml (range 8.5 ml to 30 ml). Consequently, several techniques have been established for the expansion of UCB-derived HSPCs [42], [43]. Expansion with cytokine mixtures in-vitro showed a 1000- fold expansion of HSPCs of PCB [42]. To our knowledge, no study evaluated the regenerative potential in vivo after PCB-derived HSPC expansion.

In conclusion, we found that cord blood of preterm infants contains a variety of HSPC subsets and they possess higher in-vitro clonogenic potential when compared to HSPCs isolated from cord blood of term infants. In our study, obstetric and perinatal complications did not influence the number and clonogenic capacity of HSPC subset in PCB and TCB. It seems feasible to isolate a sufficient cell count and fully functional HSPCs from PCB. Further analyses need to be done, especially the ex vivo expansion of PCB derived HSPCs and their in vivo regenerative potential. However, our findings suggest that PCB might be a potential source of HSPCs for stem cell therapy in preterm neonates.
